# Deep learning in sex estimation from a peripheral quantitative computed tomography scan of the fourth lumbar vertebra—a proof-of-concept study

**DOI:** 10.1007/s12024-023-00586-6

**Published:** 2023-02-11

**Authors:** Petteri Oura, Niina Korpinen, Allison L. Machnicki, Juho-Antti Junno

**Affiliations:** 1 Department of Forensic Medicine, Faculty of Medicine, University of Helsinki, P.O. Box 21, Helsinki, 00014 Finland; 2 Forensic Medicine Unit, Finnish Institute for Health and Welfare, Helsinki, Finland; 3 Faculty of Medicine, Research Unit of Health Sciences and Technology, University of Oulu, Oulu, Finland; 4 Department of Archaeology, Faculty of Humanities, University of Oulu, Oulu, Finland; 5 Center for Functional Anatomy and Evolution, Johns Hopkins School of Medicine, Baltimore, MD USA; 6 Cancer and Translational Medicine Research Unit, Faculty of Medicine, University of Oulu, Oulu, Finland; 7 Archaelogy, Faculty of Arts, University of Helsinki, Helsinki, Finland

**Keywords:** Sex estimation; Vertebra, Lumbar spine, Peripheral quantitative computed tomography, Imaging; Artificial intelligence, Deep learning; Forensic anthropology, Terry collection

## Abstract

Sex estimation is a key element in the analysis of unknown skeletal remains. The vertebrae display clear sex discrepancy and have proven accurate in conventional morphometric sex estimation. This proof-of-concept study aimed to investigate the possibility to develop a deep learning algorithm for sex estimation even from a single peripheral quantitative computed tomography (pQCT) slice of the fourth lumbar vertebra (L4). The study utilized a total of 117 vertebrae from the Terry Anatomical Collection. There were 58 male and 59 female cadavers, all of the white ethnicity, with the average age at death 49 years and a range of 24 to 77 years. A coronal pQCT scan was taken from the midway of the L4 corpus. Sex estimation was performed in a total of 19 neural network architectures implemented in the *AIDeveloper* software. Of the explored architectures, a *LeNet5*-based algorithm reached the highest accuracy of 86.4% in the test set. Sex-specific classification rates were 90.9% among males and 81.8% among females. This preliminary finding advances the field by encouraging and directing future research on artificial intelligence-based methods in sex estimation from individual skeletal traits such as the vertebrae. Combining quickly obtained imaging data with automated deep learning algorithms may establish a valuable pipeline for forensic anthropology and provide aid when combined with traditional methods.

**Supplementary Information:**

The online version contains supplementary material available at 10.1007/s12024-023-00586-6.

## Introduction

Sex estimation, i.e., estimating the biological genotype of an individual [1], remains a key element in the analysis of unknown skeletal remains of human origin [2, 3]. Of help is the fact that skeletal features such as bone size, shape, and structure are subject to varying levels of sex discrepancy [4, 5]. A number of skeletal sites, such as the cranium, pelvis, and extremities, have been tested in sex estimation using conventional morphometric methods. Classification rates have varied widely from ~ 40 to ~ 100% [2, 6]; however, comparisons between studies are complicated by differences in the study population, skeletal site, and sex estimation method used. An acceptable threshold for an accurate sexing tool is considered to be 80 to 95% [2, 7].

In a wide range of forensic scenarios, e.g., severe trauma, burns, and other fragmentary conditions, a limited number of skeletal elements or fragments may be recovered for examination. These cases in particular may benefit from novel sex estimation methods. The vertebrae display clear sex discrepancy in the adult spine [8] and have proven accurate in conventional morphometric sex estimation (classification rates ~ 85 to ~ 90%, maximum 94.5%) [9–20]. However, as conventional osteology methods may be laborious and only able to focus on a limited number of parameters at a time, the application of modern image recognition techniques may increase accuracy and expedite processes.

Artificial intelligence has the aim of mimicking cognitive functions by means of trained algorithms [21]. Deep learning uses neural networks in tasks such as image recognition and classification [22–24]. This is particularly beneficial in complex datasets such as those used in sex estimation from vertebral imaging data. To date, however, we only found one study that applied deep learning in this context. In their study, Malatong et al. [25] analyzed the first through fifth lumbar vertebrae (L1—L5) in a sample of 220 Thai individuals. The classification rate of a deep learning-based algorithm (92.5%) exceeded that of a digital caliper-based method (82.7%) and a morphometric image analysis method (90.0%). The findings show promise but are preliminary in nature; sex estimation methods are mostly population-specific and algorithms need external validation in large datasets. Future research is thus clearly needed. To bring additional benefit over conventional osteoarcheological methods, artificial intelligence and imaging-based sex estimation processes should be portable, quick, and automated.

With these considerations, this proof-of-concept study aimed to develop a deep learning algorithm for sex estimation from a single midcoronal peripheral quantitative computed tomography (pQCT) slice of the fourth lumbar vertebra (L4). The study utilized a total of 117 L4 vertebrae from the Terry Anatomical Collection [26].

## Materials and methods

### Material

**Fig. 1:**
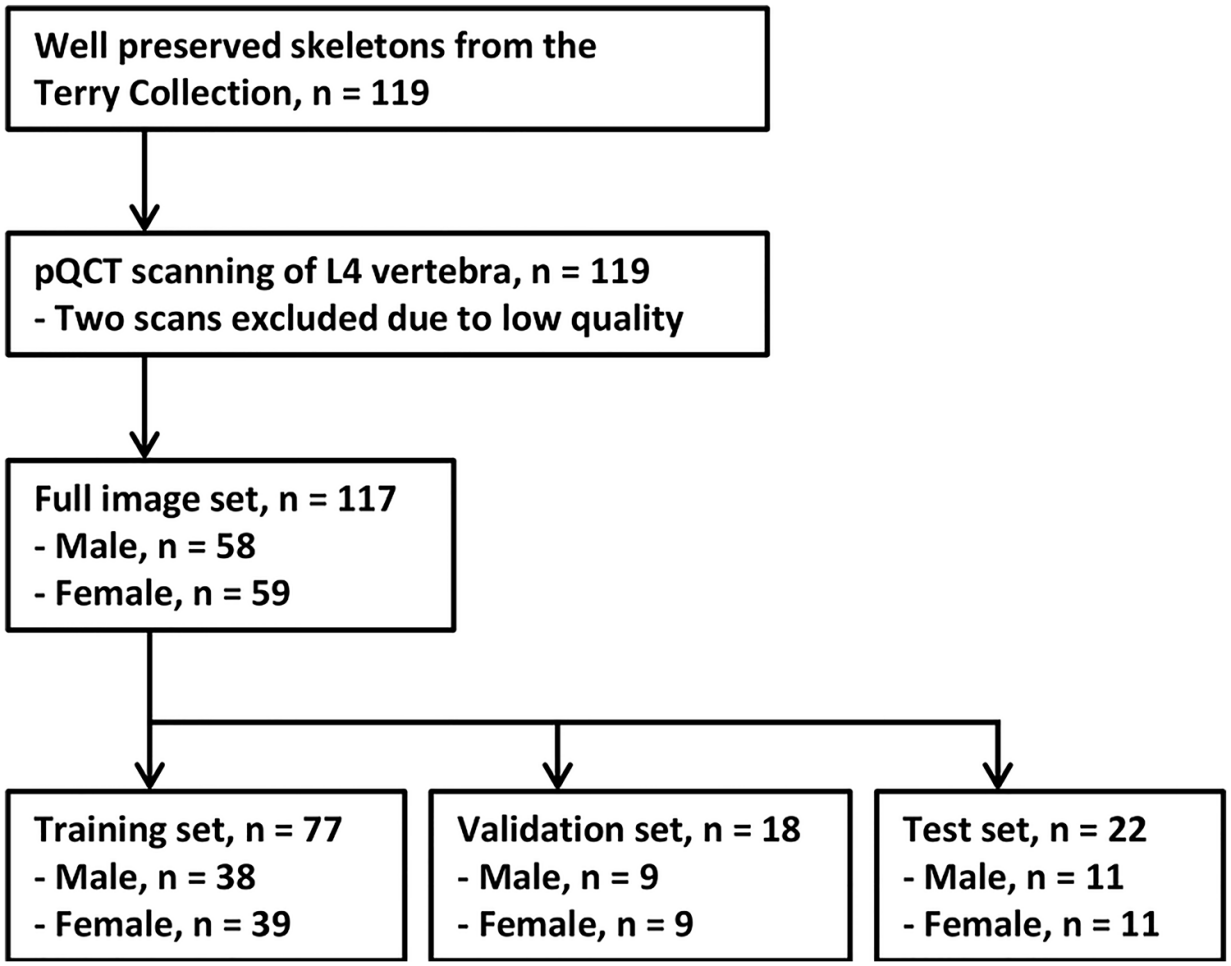
Flow-chart of the study (pQCT denotes peripheral quantitative computed tomography)

The Robert J. Terry Anatomical Collection is maintained by the Department of Anthropology, National Museum of Natural History, Smithsonian Institution. The collection originates from the late nineteenth and early twentieth century and comprises the early industrial working class population of Anglo and African origins [26]. To conduct this study, L4 vertebrae from 119 well-preserved skeletons were selected to undergo pQCT scanning (Fig. 1). As two scans were later excluded due to low quality, the final sample consisted of 117 scans. There were 58 male and 59 female cadavers, all of whom were ethnically identified as white. They had died in the twentieth century at an average age of 49 years (range 24 to 77 years).

### Peripheral quantitative computed tomography

pQCT scans of L4 were obtained using a Stratec XCT Research SA scanner and the corresponding software version 6.20 (Stratec Medizintechnik GmbH, Pforzheim, Germany). Slice thickness was set at 0.7 mm and pixel size 0.1 mm. L4 vertebrae were scanned through the vertebral corpus in a coronal plane, at the anteroposterior midpoint of the corpus. This slice was selected as it follows the primary direction of vertebral loading in humans [27, 28]. The scans were obtained by a member of the research group (J.-A.J.) who was blinded to the sex and other background information of the cadavers. Major vertebral pathologies such as crushing, wedging, osteoarthritis, and osteophyte formation were visually ruled out prior to scanning.

### Image curation

The midcoronal pQCT slices of L4 were exported as BMP files and opened in Photoshop Elements version 2022 (Adobe, San Jose, CA, USA). The vertebrae were systematically extracted with a rectangular cutter following a ratio of approximately 175 × 110 pixels. The images were ensured to be in grayscale format and contrast was systematically adjusted with the “Auto Contrast” tool. The curated images were saved in GIF format. Image curation was performed by a member of the research group (P.O.) who was blinded to the sex and other background information of the cadavers. Please see Supplementary Fig. S1 for samples of curated images.

### Deep learning procedure

*AIDeveloper* software version 0.1.2 [24] was used for the deep learning procedure (i.e., training, validation, and testing of algorithms). *AIDeveloper* is an open-source deep learning software that has a number of neural network architectures available for image classification.

First, the curated images were randomly assigned to training, validation, and test sets, following an approximate 70%–15%–15% ratio in the division (Fig. 1). A random number generator tool of SPSS Statistics version 26 (IBM, Armonk, NY, USA) was used in the randomization. Then, the training and validation image sets were uploaded to *AIDeveloper*, and training and validation rounds were run. A list of the explored network architectures is given in Table 1; they were selected as they were readily implemented in the software package. A detailed configuration of the settings and parameters is given in Supplementary Table S1. The best algorithm from each architecture was taken forward to the testing round which used the test image set as material.

**Table 1:** List of explored network architectures and performance metrics of best models

Neural network architecture	Training and validation set			Testing set	
	Best model	Training accuracy	Validation accuracy	Testing accuracy	Correct per class (male/female, %)
LeNet5	13	0.83	0.77	0.86	90.9/81.8
MLP_64_32_16	18	0.72	0.83	0.77	63.6/90.9
MLP_16_8_16	118	1.00	0.73	0.77	90.9/63.6
MLP_8_8_8	76	0.72	0.80	0.73	54.5/90.9
LeNet5_bn_do	41	0.89	0.93	0.68	90.9/45.5
MLP_24_16_24_skipcon	39	0.83	0.90	0.68	45.5/90.9
MhNet1_bn_do_skipcon	1	0.60	0.87	0.68	100.0/36.4
LeNet5_do	28	0.73	0.83	0.68	90.9/45.5
MLP_64_80_32	42	0.93	0.80	0.68	45.5/90.9
MLP_72_64_48_48	29	0.75	0.80	0.68	45.5/90.9
MLP_256_128_64_do	148	0.76	0.70	0.68	45.5/90.9
MLP_24_16_24	24	0.79	0.80	0.63	36.4/90.9
MhNet2_bn_do_skipcon	1	0.61	0.87	0.59	90.9/27.3
MLP_4_4_4	13	0.53	0.57	0.59	100.0/18.2
MLP_72_48_24_32	74	0.98	0.83	0.55	36.4/72.7
TinyCNN	1448	0.91	0.83	0.55	90.9/18.2
TinyResNet	44	0.65	0.73	0.55	90.9/18.2
MLP_72_80_32	199	0.94	0.80	0.50	0.0/100.0
LeNet5_bn_do_skipcon	156	0.87	0.83	0.45	72.7/18.2

An a priori decision was made to evaluate the algorithms based on the following performance metrics [24, 29]:

- Accuracy (i.e., true positives + true negatives)/total)
- Precision (i.e., true positives/(true positives + false positives))
- Recall (i.e., true positives/(true positives + false negatives))
- F1 value (i.e., 2 × (precision × recall)/(precision + recall))

## Results

A total of 117 L4 images were used in this study. The images were divided into training (65.8%), validation (15.4%), and test sets (18.8%) (Fig. 1).

Table 1 lists the performance metrics of the explored algorithms. Classification rates in the training and validation sets ranged from 0.53 to 1.00 and from 0.57 to 0.93, respectively. Testing accuracies were generally lower than training and validation accuracies, ranging from 0.45 to 0.86.

A *LeNet5*-based algorithm reached the highest overall testing accuracy, classifying 90.9% of males and 81.8% of females correctly (Tables 2 and 3). The neural network structure of the algorithm is given in Supplementary Table S2, and a visual overview of its performance in the test image set is shown in Supplementary Fig. S1.

**Table 2:** True and predicted classes on test set data according to the best algorithm (*LeNet5*)

	Predicted class	
	Class 0 (male)	Class 1 (female)
True class		
Class 0 (male)	90.9% (10/11)	9.1% (1/11)
Class 1 (female)	18.2% (2/11)	81.8% (9/11)

**Table 3:** Performance metrics of the best algorithm on test set data (*LeNet5*)

Metric	Class 0 (male)	Class 1 (female)
Testing accuracy	0.86	0.86
F1	0.87	0.86
Precision	0.83	0.90
Recall	0.91	0.82

## Discussion

This preliminary study proved the concept of using a single pQCT slice of the L4 vertebra in artificial intelligence-based sex estimation. Altogether 19 neural network architectures were explored and their performance varied significantly. The best algorithm estimated sex correctly in 86.4% of the test set cases; sex-specific classification rates were 90.9% among males and 81.8% among females.

Most previous literature has described conventional morphometric methods to estimate sex from the vertebrae. Classification rates have mainly fluctuated between 85 and 90% [9–20], and the accuracy of the current best algorithm also falls within this range. In comparison to a previous Thai study [25] that developed a deep learning algorithm for sex estimation from L1 to L5, the accuracy was somewhat lower (86.4% vs. 92.5%). However, the present algorithm was developed on the basis of one vertebra only (L4), and both external shape and internal morphology were captured in the midcoronal scans. In contrast, the Thai study was mostly based on surface plate images and external morphology. Moreover, comparisons between studies should be made with caution, bearing in mind differences in source population, sample size and heterogeneity, vertebral parameters, and data analysis methods. Of note is the fact that the current best algorithm clearly exceeded the 80% threshold for an acceptable sexing tool, encouraging further research on the topic.

This preliminary study explored 19 neural network architectures that were readily implemented in the deep learning software. There were little prior data and thus no a priori hypotheses regarding their performance relative to the study question. Notably, the performance of individual architectures varied significantly from the equivalent of a coin toss to the level of an acceptable sexing tool. While the authors believe that the differences are primarily explained by technical differences between architectures, future studies are required to investigate further. In the current dataset, some *LeNet5*- and multilayer perceptron-based architectures appeared to perform best.

As conventional osteology methods may be laborious and able to focus on a limited number of parameters at a time, novel sex estimation methods should be portable, quick, and automated. Despite being a three-dimensional imaging modality with an accurate depiction of bone geometry and microstructure, pQCT is designed for compactness and portability [30]. It is also swift to operate. In forensic scenarios, such as mass disasters in remote areas, the portability of the equipment and diagnostic devices would be a key advantage. Combining quickly obtained imaging data with automated image recognition algorithms may establish a valuable pipeline for forensic anthropology. Automated image classification also reduces subjectivity and measurement errors [25]. Naturally, large datasets and careful external validation will be necessary.

Several aspects require clarification in the future. First, studies should aim to explore whether the axial, sagittal, or coronal plane carries the most information value in sex estimation and compare these planes to a true three-dimensional profile of the vertebra. In a Finnish study using conventional metric methods, vertebral width and depth had clearly higher predictive values than vertebral height in all studied age groups [12]. It would also be interesting to explore whether geometry or microstructure possesses higher information value in sex estimation. Second, the drivers of misclassification should be identified. Males have generally larger vertebrae than females [8]; however, several lifestyle-related factors may influence the vertebrae and increase inter-individual variation in vertebral geometry and microstructure (e.g., age [12, 31], body size [32, 33], physical activity [34, 35], nutrition [36, 37]). Although major vertebral pathologies were ruled out, degenerative changes may also have affected the sex estimation accuracy. Third, robust algorithms should be based on large, representative, and contemporary samples. Careful external validation will be required prior to routine forensic use. On the basis of the current results, a similar pipeline utilizing post-mortem CT scans could be achievable; however, larger spinal segments or other skeletal elements should be explored as material, as they may well outperform L4. Naturally, careful optimization and validation would be necessary prior to routine implementation.

The main strength of the study was a novel approach, using a single pQCT slice of L4 in artificial intelligence-based sex estimation. Importantly, the best algorithm reached an accuracy of 86.4%. The proof-of-concept aim of the study was fulfilled. The main limitations were a relatively small sample size, wide age range, and lack of external validation. Although inter-observer bias cannot be fully ruled out, manual work was only required in few strictly standardized steps during the process (i.e., positioning of the vertebra for pQCT and image curation). However, in future applications, the pipeline would ideally be fully automated.

The lack of transparency remains a great limitation of artificial intelligence-based methods, especially in the legal context. As a standalone concept, artificial intelligence lacks reliability, since it may be subject to varying kinds of bias, but cannot be examined similarly to a human witness in the courtroom [38]. The authors, therefore, suggest that algorithms should primarily serve as additional tools for forensic anthropologists. The final interpretation of a case would always remain with the expert, preferably combining artificial intelligence-based tools with conventional evidence.

## Conclusion

In this study of 117 midcoronal pQCT slices of the L4 vertebra, a *LeNet5*-based deep learning algorithm reached a sex estimation accuracy of 86.4%. This preliminary finding advances the field by encouraging and directing future research on artificial intelligence-based methods in sex estimation from individual skeletal elements such as vertebrae. Combining quickly obtained imaging data with automated deep learning algorithms may establish a valuable pipeline for forensic anthropology and provide aid when combined with traditional methods.

## Key points

1. The vertebrae display clear sex discrepancy and have proven accurate in conventional morphometric sex estimation.
2. This study tested the application of several deep learning algorithms for sex estimation from a single computed tomography slice of the fourth lumbar vertebra (L4).
3. A *LeNet5*-based algorithm reached the highest accuracy of 86.4% in the test set.
4. This finding encourages future studies on artificial intelligence methods in sex estimation from individual skeletal traits such as the vertebrae.

### Supplementary Information

Below is the link to the electronic supplementary material.

Supplementary file1 (DOCX 3290 KB)

Supplementary file2 (DOCX 19 KB)

## Data Availability

The datasets generated and analyzed during the study are available from the corresponding author upon reasonable request.
