# Supplementary material for: Deep learning in sex estimation from a peripheral quantitative computed tomography scan of the fourth lumbar vertebra—a proof-of-concept study
Source: Forensic Sci Med Pathol. 2023 Feb 11;19(4):534–40. doi: 10.1007/s12024-023-00586-6 (PMC10752832; doi:10.1007/s12024-023-00586-6)
Supplement: Supplementary file 1 — Supplementary file1 (DOCX 3290 KB) [file 12024_2023_586_MOESM1_ESM.docx]

**SUPPLEMENTARY FIGURES**





**Supplementary Figure 1**. Classification of test set cases by the best algorithm.
