# Supplementary material for: Deep learning in sex estimation from a peripheral quantitative computed tomography scan of the fourth lumbar vertebra—a proof-of-concept study
Source: Forensic Sci Med Pathol. 2023 Feb 11;19(4):534–40. doi: 10.1007/s12024-023-00586-6 (PMC10752832; doi:10.1007/s12024-023-00586-6)
Supplement: Supplementary file 2 — Supplementary file2 (DOCX 19 KB) [file 12024_2023_586_MOESM2_ESM.docx]

**SUPPLEMENTARY TABLES**

**Supplementary Table 1**. Parameters of the neural network training process in *AIDeveloper*.

| Parameter | Value |
| --- | --- |
| Model specification |  |
| Network architecture | Several tested, please refer to **Table 1**. |
| Input image size (pixels) | 100 x 100 |
| Image normalization | Division by 255 |
| Color mode | Grayscale |
| Padding | No |
| Total number of epochs | 2000 |
| Events per epoch in training set (per class) | 50 |
| Events per epoch in validation set (per class) | 15 |
| Image augmentation |  |
| Vertical flip | No |
| Horizontal flip | No |
| Rotation (degrees, range) | -3…3 |
| Width shift (%, range) | -0.001…0.001 |
| Height shift (%, range) | -0.001…0.001 |
| Zoom (%, range) | -0.001…0.001 |
| Shear (%, range) | -0.005…0.005 |
| Brightness by multiplication (coefficient, range) | 0.70…1.30 |
| Contrast (coefficient, range) | 0.70…1.30 |
| Gaussian noise (mean with standard deviation) | 0.00 (3.00) |
| Blurring (kernel size, range) | 0…5 |
| Number of epochs after which refreshes | 1 |

**Supplementary Table 2**. Neural network structure of the best architecture (*Lenet5*).

| # | Layer | Output Shape | Parameters |
| --- | --- | --- | --- |
| 1 | inputTensor | None, 96, 96, 6 | 156 |
| 2 | activation_1 | None, 96, 96, 6 | 0 |
| 3 | max_pooling2d_1 | None, 48, 48, 6 | 0 |
| 4 | conv2d_1 | None, 44, 44, 16 | 2416 |
| 5 | activation_2 | None, 44, 44, 16 | 0 |
| 6 | max_pooling2d_2 | None, 22, 22, 16 | 0 |
| 7 | flatten_1 | None, 7744 | 0 |
| 8 | dense_1 | None, 120 | 929 400 |
| 9 | activation_3 | None, 120 | 0 |
| 10 | dense_2 | None, 84 | 10 164 |
| 11 | activation_4 | None, 84 | 0 |
| 12 | dense_3 | None, 2 | 170 |
| 13 | outputTensor | None, 2 | 0 |

Total parameters *n* = 942 306, trainable parameters *n* = 942 306, non-trainable parameters *n* = 0
